# The Xomed Monopolar Cranial Nerve Stimulator Electrode: A Surprising Handy Tool for Deep Dissection of Epidermoid Tumors

**DOI:** 10.7759/cureus.2058

**Published:** 2018-01-12

**Authors:** Gopalakrishnan M Sasidharan

**Affiliations:** 1 Department of Neurosurgery, Jawaharlal Institute of Postgraduate Medical Education and Research (JIPMER), Puducherry, India.

**Keywords:** epidermoid cyst, cerebellopontine angle, intraoperative physiological monitoring, electromyography, iatrogenic injury, cranial nerve palsy, intraoperative neuromonitoring

## Abstract

Epidermoid cysts are notorious for their propensity to sneak into deep recesses between cranial nerves in the posterior fossa. Attempts to achieve complete excision using ordinary instruments when tempted by the seeming ease of dissection is known to cause unacceptable deficits. The Xomed monopolar stimulator electrode probe of the nerve integrity electromyography monitor has several advantages when used as the primary dissection tool for deep-seated epidermoid cysts. Cerebellopontine angle epidermoid is the classical prototype of a strategically placed deep-seated epidermoid tumor.

The author describes the use of the monopolar stimulator electrode of the nerve integrity electromyography monitor as the primary dissection tool for excising epidermoid cysts of the cerebellopontine angle. Thin profile, rounded nontraumatic tip, and springiness of the body of the monopolar electrode aid the dissection.

The monopolar electrode was used to tease and scoop out the flakes aided by a fine suction. An initial internal decompression allowed the capsule to be folded away and separated from neurovascular structures using the dissector. The thin profile of both the instruments allowed good visualization and delicate control over the dissection in depths of the resection cavity without undue traction or impacts on superficially dissected cranial nerves. This novel use of the monopolar electrode was employed in the surgical excision of epidermoid cysts of the cerebellopontine angle in nine patients.

Total or near total excision was possible in eight of the nine patients who underwent excision using this technique. In three patients, mild deficits primarily of the fifth nerve sensory function were noted.

The Xomed monopolar stimulating electrode of the nerve integrity electromyography monitor is an ideal instrument for deep dissection of epidermoid cysts in areas where neurovascular structures are at risk.

## Introduction

Epidermoid tumors are known for their ability to insinuate among neurovascular tissues and expand into deeply cavernous recesses around the cerebrum and brainstem. These pearly beauties are so notorious for their ability to seduce even good surgeons into following them to places where many dangers lurk, that they can truly be called as the sirens among brain tumors. Their waxy friable nature and the fact that these desquamated clumps of tissue do not bleed at all tempt surgeons into pursuing them for that elusive goal of total resection- similar to the fate that sailors meet when they listen to the irresistibly sweet songs of the beautiful sirens of the high seas.

Complications of surgery of posterior fossa epidermoid cysts may be highly under-reported and might exceed the reported rates of postoperative morbidity of 13.6% and mortality of 8.9% [1]. Despite the fact that literature is replete with warnings of severe deficits even in the hands of high surgical volume experts, patients continue to get injured when surgeons pursue the temptation of a complete resection of adherent epidermoid cysts [2-3].

## Technical report

Surgical technique

**Figure 1:**
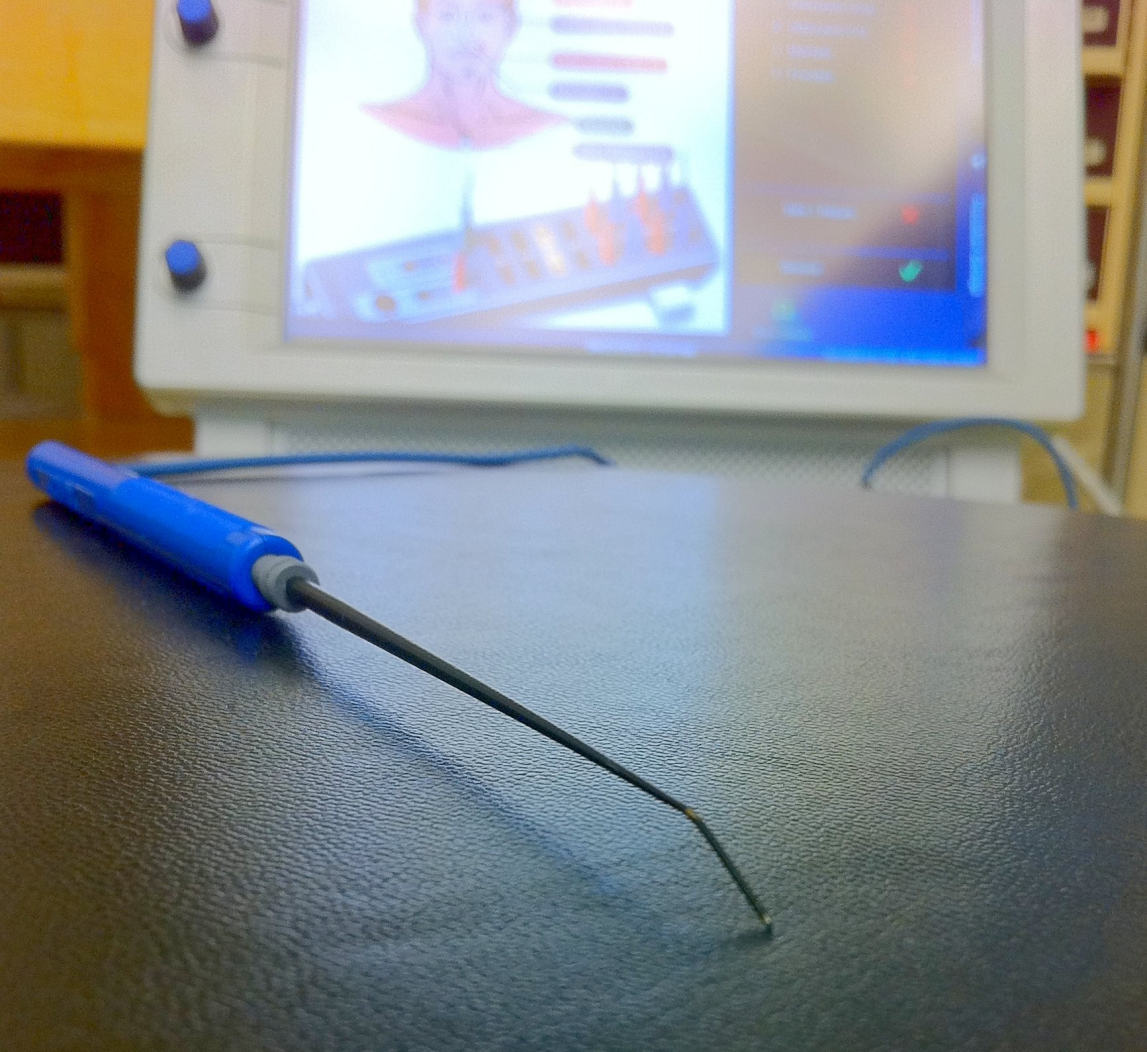
'The epidermoid dissector' The monopolar electromyography stimulator electrode probe. Note the blunt point of its slender malleable body. It was used without any modifications. The cranial nerve integrity monitor is seen in the background.

The cranial nerve integrity monitor (NIM Neuro 3.0, Medtronics Inc, Minneapolis) was used for monitoring the cranial nerves during excision of posterior fossa epidermoid in the region of the cerebellopontine angle. The monopolar stimulating electrode probe was used as the primary tool for dissecting the tumors in nine patients with cerebellopontine angle based epidermoid tumors that the author had operated between 2011 and 2014 (Figure 1). 

All patients were operated in a supine oblique position using a retro-mastoid craniectomy to approach the lesion. The fifth, seventh and lower cranial nerves were routinely monitored using the nerve integrity monitor with leads placed in the orbicularis oris, orbicularis oculi, masseter and trapezius muscles. Muscle relaxants were discontinued to enable cranial nerve monitoring after induction of anesthesia. An initial stimulator current setting of 1.0 mA was used and it was reduced progressively if the current spread was causing stimulation of cranial nerves from too far away.

**Figure 2:**
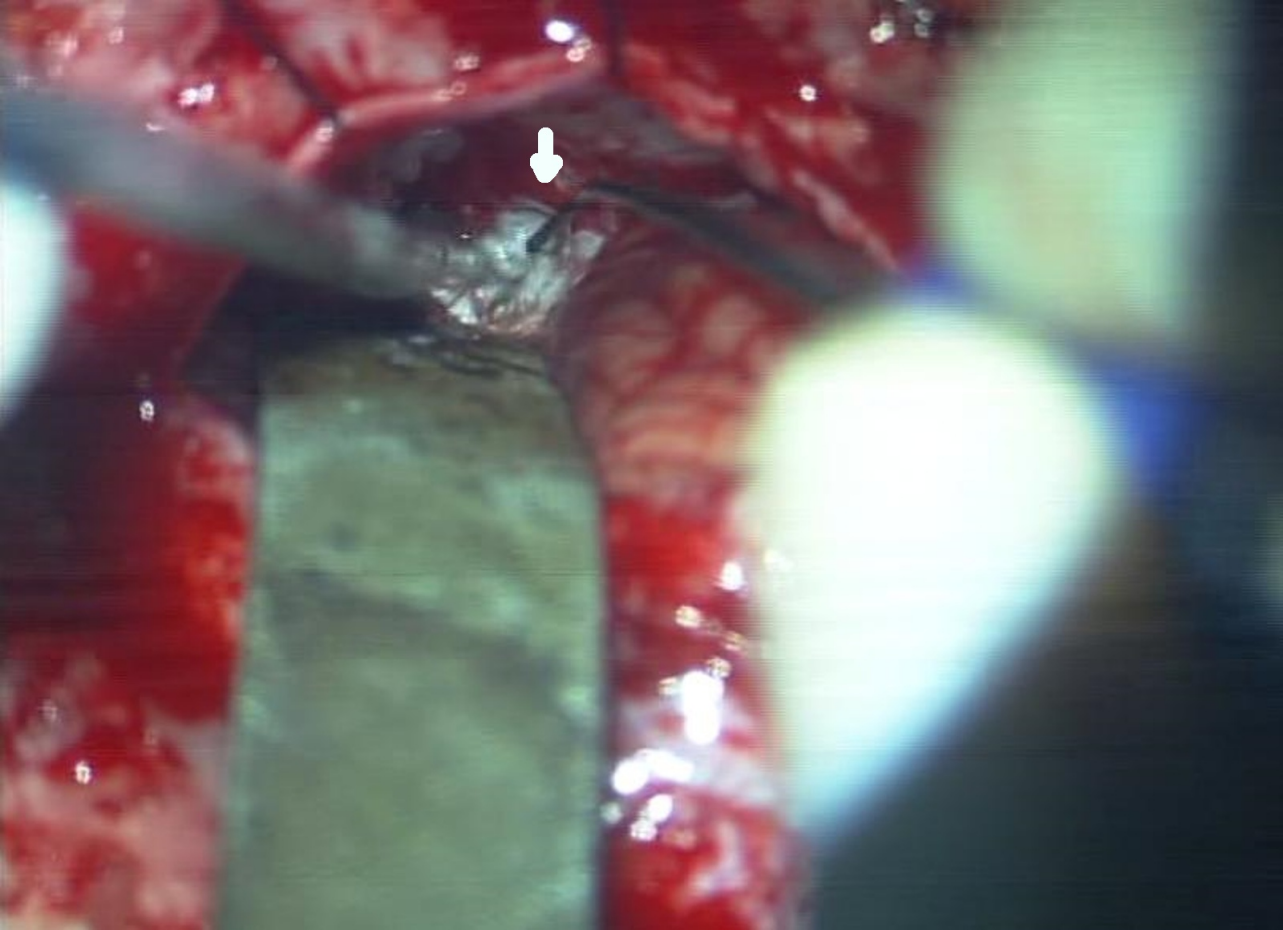
The view through the operating microscope The view through the operating microscope shows the stimulator-dissector on the right side (arrow). The slender size of the ‘epidermoid dissector’ is obvious in comparison to the size 1 French suction cannula on the left.

After a totally shave-less, retro-mastoid craniectomy, the dura was opened a few millimeters away from the dural venous sinuses, leaving the dural flap to lie over the exposed cerebellar surface to protect it. Flakes of the epidermoid cyst were removed by gently teasing out the clumps with the rounded tip of the monopolar electrode probe and by suctioning out the material disengaged by the maneuver using finely controlled size 1 French suction cannula (Day Bailey locking suction, Mizhuo Medical Inc, Tokyo) (Figure 2). 

As the impacted material is decompressed, the gossamer-thin cyst capsule is also removed by using the electrode probe to separate and fold it away from adherent structures using the leverage provided by the thin residual layer of cyst content adherent to the inside of the capsule. Fragments of the cyst wall that were too adherent to be removed by this technique were left behind instead of aggressively pursuing larger instruments like micro-forceps. Gentle retraction of the cerebellar hemispheres was used to prevent the tumor cavity from collapsing and impairing visualization as the cyst contents were progressively removed. Most of the visualization was done by dynamically retracting the overhanging cerebellar hemisphere using the body of the fine suction cannula. While dissecting between cranial nerves in various nooks and corners, particular attention was directed to audible alarms generated by the monitor due to stimulation of nearby cranial nerves to facilitate changing the trajectory or force of use of the dissector. Repetitive stimulation of cranial nerves during the course of the surgery was not found to cause any adverse effects. In two patients, a thirty-degree endoscope was used to visualize bits of the tumor which were initially missed out in the initial excision. Excision was always carried out under operating microscope magnification only. The superior petrosal vein was always preserved.

## Discussion

Typically, injuries to neurovascular structures occur in three situations especially at the very end of the operation for epidermoid tumors.

While trying to control sudden and unexpected bleeding from an injured vein in a deep operative cavity which quickly obscures visualization of once cleanly dissected nerves.

Relatively large dissectors or tumor-holding forceps stretching out or directly impacting superficially dissected out nerves and vessels while the surgeon works between the latter structures to reach deeper areas under high magnification. Structures that are superficial are rather out of focus at such magnifications.

Rough dissection of the densely adherent capsule, especially from the surface of a burrowed out cavity in the brainstem.

Cranial nerve monitoring is routinely used in surgeries of the posterior fossa for identifying and preserving nerves. Here, the author describes his experience of using the monopolar stimulator (Medtronic Xomed TM, Minneapolis) of the NIM 3.0 electromyogram monitor itself as a dissecting tool in such circumstances. 

The NIM 3.0 Monopolar stimulator is uniquely useful for dissecting out an epidermoid because of its characteristics although the manufacturers did not intend it for this particular use.

The angulated tip of the monopolar stimulator is small, measuring just less than a millimeter yet it has a non-traumatic point. Its body has a certain springiness to it and is flexible to an extent giving tactile feedback to the surgeon’s fingers that other instruments cannot provide. Even in very deep cavities (7 cm and beyond), the tactile feedback obtained when encountering an incompletely dissected out vessel or neural structure is distinctly different from the feel of the give-way sensation one gets when a lump of epidermoid is teased out. This confers a measure of safety that one would not get when using even the finest dissectors which are not so flexible and light. When used as a dissector, the monopolar dissector allows exquisitely precise and gentle manipulation of critically important structures at a depth.

Another advantage is the relatively low cross-section of its body that gives much room when working between cranial nerves, especially in the posterior fossa. Since it is by design a monopolar cranial nerve stimulator, injury of cranial nerves by inadvertent overstretching is minimized by the audio alarms the machine would give off as soon as one touches superficially situated structures while angulating its tip deep in the tumor cavity.

**Figure 3:**
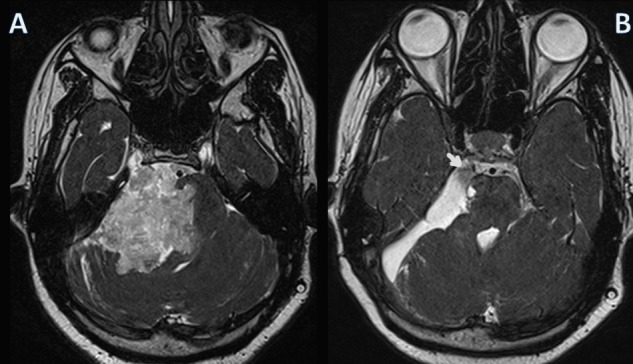
Preoperative and postoperative magnetic resonance imaging (MRI) Heavily T2-weighted MRI with thin slices (constructive interference in steady state) shows the preoperative lesion that had dug itself into the pontine surface (A). The postoperative MRI was taken three months after the surgery and it shows complete excision of the tumor (B). A flow related hypo-intensity is seen in the cerebrospinal fluid collection close to the fifth nerve (arrow). Removing the part of the tumor that was insinuating into the pontine surface caused mild and transient numbness in the trigeminal nerve distribution in the immediate postoperative period.

The author uses a combination of finely controlled suction in a size 1F cannula (Day Bailey locking suction, Mizhuo Medical Inc, Tokyo) to suck out the friable impacted flakes and the monopolar electrode to dissect away the thin capsule and lumps of epidermoid flakes from neurovascular structures. Any part of the capsule that is too tightly adherent that it cannot be dissected away with this dissector might well be wisely left behind to avoid unacceptable deficits. The rate of symptomatic recurrence of sub-totally or near-totally resected epidermoids may not be significantly different from total excised ones [4] and tend to occur a decade later when safe re-excision can be offered again [5]. Hence the overarching goal of surgery of epidermoid cyst should be to achieve a good decompression while minimizing the possibility of inadvertent neurovascular injury. This instrument helps achieve that objective (Figure 3).

## Conclusions

The easily dissectible but enveloping nature of the epidermoid is quite different from any other tumor and warrants a dissector that is fine yet non-traumatic, springy and light. Curiously, the Xomed NIM 3.0 monopolar stimulator electrode fits the bill and has the added advantage that it can continuously monitor cranial nerves at risk during surgery for posterior fossa epidermoid cysts. The author recommends the use of this dissector in all operations of posterior fossa epidermoid cysts and would even advice ordering the monopolar stimulator electrode alone or fashioning an instrument of similar characteristics if the NIM 3.0 monitor is not available in a resource-constrained setting.
